# Association of White Matter Hyperintensities With Serum Lipid Profile and Hematologic Parameters in Migraine Patients

**DOI:** 10.7759/cureus.70452

**Published:** 2024-09-29

**Authors:** Firdevs Ezgi Ucan Tokuc, Ruhsen Ocal

**Affiliations:** 1 Neurology, Antalya Training and Research Hospital, Antalya, TUR

**Keywords:** aura, hdl-c, ldl-c, migraine, plr, white matter hyperintensity

## Abstract

Introduction

Migraine is one of the most common neurological diseases. Comorbidities, especially stroke, may be observed. White matter hyperintensities are common in migraine patients. Although many factors have been associated with white matter hyperintensities, such as age, gender, migraine type, frequency, and presence of aura, the etiology of white matter hyperintensities remains unclear. We aimed to study the relationship between the development of white matter hyperintensities and serum lipid profiles and hematologic parameters in migraine patients.

Methods

Demographic data, comorbidities, migraine types, presence of aura, and frequency of attacks were reviewed. Cranial MRI scans, hematologic profiles, and lipid profiles were analyzed.

Results

The study included 51 patients with white matter hyperintensity on cranial MRI and 76 migraine patients with normal cranial MRI. High-density lipoprotein cholesterol (HDL-c) was statistically significantly higher in the patients with white matter hyperintensity in the cranial MRI (WMH+) group. A statistically significant cut-off value was found for the PLR parameter in differentiating white matter hyperintensity on cranial MRI in migraine patients.

Conclusion

In our study, we examined the factors contributing to the development of white matter hyperintensity in migraine patients and observed the correlation between disease duration and white matter hyperintensity. While no correlation was observed between low-density lipoprotein cholesterol (LDL-c) and white matter hyperintensity, HDL-c was dramatically elevated in patients with white matter hyperintensity. Furthermore, our study shows that PLR is a useful, easily accessible, and practical parameter for detecting the development of white matter hyperintensity in migraine patients. Larger-scale randomized studies are needed for a clearer interpretation of these findings.

## Introduction

Migraine is one of the most common neurologic diseases and is a recurrent primary headache disorder that may be accompanied by transient neurologic symptoms known as aura [1]. Being more common in young people and women, it greatly affects the daily life activities of patients. In addition, an increased risk of comorbid diseases such as cardiovascular disease, stroke, and dementia has been demonstrated in migraine patients [2-4].

White matter hyperintensity (WMH) are small punctate lesions that appear hyperintense in T2-weighted and fluid-attenuated inversion recovery (FLAIR) sequences, especially in the deep or periventricular white matter and are caused by damage to the small vessels of the brain [5,6]. Factors such as disruption in the blood-brain barrier, neuroinflammation, and impaired cerebral blood flow are involved in its pathogenesis, and its prevalence increases with age, especially in people over 50 [6,7]. However, it is well known that the incidence of WMH is 2 to 4 times higher in migraine patients compared to the healthy population. Also, migraine alone has been reported to be an independent risk factor in the development of WMH in the population under the age of 50 years without any cardiovascular risk factor [8,9]. Although the pathophysiological mechanism is not clear, many factors, such as ischemic damage due to metalloproteinases induced by cortical spreading depolarization, microemboli, hypercoagulability, and endothelial dysfunction, are discussed [9]. Although it has been associated with many factors, including age, gender, migraine type, frequency, and presence of aura in some previous studies, the etiology of WMH still remains unclear [10-12]. Nevertheless, it is well known that neuroinflammation constitutes the significant pathophysiologic basis of WMH development [13,14].

In particular, it is generally recognized that patients with migraine with aura have a higher cardiovascular risk profile than individuals without migraine. Despite this, the association of white matter lesions with vascular risk factors such as hypercholesterolemia and diabetes has not been proven [15-17].

Based on this contradictory and uncertain etiology in the development of WMH, we aimed to study the relationship between the development of WMH and serum lipid profiles and hematologic parameters in migraine patients.

## Materials and methods

Study population and design

The study was approved by the ethics committee of Antalya Training and Research Hospital (25/4/2024-5/11) and was designed in accordance with the ethical standards of the Declaration of Helsinki.

Patients over the age of 18 and under the age of 50 who met the criteria for migraine according to the International Headache Disorders Classification-III (beta version) and underwent cranial magnetic resonance imaging (MRI) between January 2022 and January 2024 at the neurology outpatient clinic of Antalya Training and Research Hospital were retrospectively reviewed [18].

All patients with a history of hyperlipidemia, hypertension, diabetes mellitus, obesity, coronary artery disease, rheumatic disease, oral contraceptive use, antilipidemic therapy use, cerebrovascular disease, and infection during the examination were excluded from the study. In addition, patients who did not have complete blood and biochemistry tests, as well as all patients whose vasculitis markers (antinuclear antibody, antineutrophil cytoplasmic autoantibody, antiphospholipid antibody, lupus anticoagulant, VDRL, C3,C4, anticardiolipin antibody) were not requested after WMH was detected or those who were requested but found to be positive for any marker were excluded from the study.

WMH were considered if visible as hyperintense on T2-weighted and FLAIR images, without hypointensity on T1-weighted scans, and were larger than 3 mm [19].

Fifty-one migraine patients with WMH on cranial MRI (WMH (+) group) and 76 migraine patients without WMH on cranial MRI (WMH (−) group) were included in the study. Demographic data, comorbidities, migraine types, presence of aura, and frequency of attacks were reviewed from the epicrisis notes of all patients. 

Laboratory assessment

Laboratory data of all patients were retrospectively reviewed, and hematologic profiles and lipid profiles were noted. In addition, neutrophil count/lymphocyte count ratio (NLR), platelet count/lymphocyte count ratio (PLR), and platelet count × neutrophil count/lymphocyte count ratio (SII) were calculated from blood values.

Statistical analysis

The data were analyzed using the IBM SPSS V23 program (IBM Corp., Armonk, NY). Compliance with normal distribution was examined with the Kolmogorov-Smirnov Test. Independent samples T-test was used to compare the parameters that fit the normal distribution according to the groups. The Mann-Whitney U test was used to compare the parameters that did not fit the normal distribution according to the groups. Binary logistic regression analysis was used to analyze the risk factors affecting WMH (+) status. ROC analysis was used to determine the cut-off value for the parameters. The analysis results were expressed as frequency (percentage) for categorical variables and mean ± standard deviation and median (minimum-maximum) for quantitative variables. The significance level was taken as p<0.050.

## Results

The study included 127 migraine patients, 51 in the WHL (+) group and 76 in the WHL (−) group. Age and duration of migraine diagnosis were statistically significantly higher in the WMH (+) group (p≤0.001, <0.001). No statistical significance was observed between the groups regarding gender, migraine type, and presence of aura. Comparing the laboratory data of both groups, the PLR was observed to be higher in the WMH (+) group (p=0.020). High-density lipoprotein cholesterol (HDL-c) was statistically significantly higher in the WMH (+) group (p=0.022) (Tables 1-2).

**Table 1:** Comparison of demographic stages and parameters according to groups WMH: white matter hyperintensity; LDL-c: low-density lipoprotein cholesterol; HDL-c: high-density lipoprotein cholesterol; NLR: neutrophil-to-lymphocyte ratio; PLR: platelet-to-lymphocyte ratio; SSI: systemic immune-inflammation index, (platelet count x neutrophil count)/lymphocyte count. *Mann Whitney U test; **Independent samples T-test; mean ± standard deviation; median (minimum-maximum).

	WMH Status		Test statistics	p-value
	WMH (−)	WMH (+)		
Age	35.82 ± 8	41.84 ± 6.16	1071	<0.001*
	36 (21–49)	43 (20–49)		
Mean frequency of headache (months)	9.83 ± 7.29	8.86 ± 7.24	1751.5	0.357*
	8 (1–30)	6 (1–30)		
Mean disease duration (years)	6.5 ± 3.71	9.39 ± 4.07	1121.5	<0.001*
	5.5 (2–20)	10 (1–20)		
Hemoglobin (g/dl)	12.97 ± 1.18	12.98 ± 1.22	1753	0.362*
	12.9 (9.4–16.3)	13.3 (9.2–15)		
Leukocyte (×10^3^/L)	7.46 ± 1.85	7.67 ± 1.79	−0.656	0.513**
	7.5 (4.2–13.9)	7.4 (5–12.9)		
Neutrophil (×10^3^/L)	4.27 ± 1.37	4.46 ± 1.3	1765	0.395*
	4 (1.4–7.1)	4.3 (2.1–7.2)		
Lymphocyte (×10^3^/L)	2.35 ± 0.65	2.29 ± 0.7	1843	0.640*
	2.3 (1.17–5.3)	2.1 (0.8–4.2)		
Platelet (×10^3^/L)	266.66 ± 59.34	289.33 ± 63.69	1506.5	0.034*
	262.5 (132–497)	277 (178–508)		
LDL-c (mg/dl)	110.76 ± 30.19	118.47 ± 37.49	−1.278	0.203**
	110 (53–187)	119 (62–226)		
HDL-c (mg/dl)	54.03 ± 12.04	59.68 ± 15.34	−2.322	0.022**
	54 (30–78)	58 (33–130)		
Triglyceride (mg/dl)	130.74 ± 89.34	115.86 ± 53.08	1894.5	0.831*
	97.5 (14–389)	100 (49–260)		
NLR	1.89 ± 0.67	2.18 ± 1.17	1712	0.266*
	1.73 (0.61–3.97)	1.91 (0.66–8.13)		
PLR	119.16 ± 33.28	136.17 ± 43.2	−2.377	0.020**
	117.68 (48.89–230.5)	129.52 (60.5–244.55)		
SSI	506.87 ± 215.05	617.02 ± 297.76	1549	0.056*
	467.9 (104.09–1083.35)	538.36 (221.16–1584.38)		

**Table 2:** Examining the relationship between groups and categorical data WMH: white matter hyperintensity *Fisher's exact test; **Yates correction; frequency (percentage)

	WMH status		Total	Test statistics	p-value
	WMH (−)	WMH (+)			
Sex					
Female	68 (58.6)	48 (41.4)	116 (91.3)	-	0.524*
Male	8 (72.7)	3 (27.3)	11 (8.7)		
Migraine type					
Episodic migraine	40 (57.1)	30 (42.9)	70 (55.1)	0.256	0.613**
Chronic migraine	36 (63.2)	21 (36.8)	57 (44.9)		
Aura					
No	67 (59.3)	46 (40.7)	113 (89)	0.005	0.944**
Yes	9 (64.3)	5 (35.7)	14 (11)		

When the risk factors affecting being WML (+) were analyzed as a univariate model, it was observed that a one-unit increase in age increased WML 1.125 times (<0.001), and when the PLR value increased by one unit, the risk of being WML (+) increased 1.012 times (p=0.017). When the SII value increases by one unit, the risk of being WML (+) increases 1.002 times (p=0.021). According to the results of the multiple models, when the triglyceride value decreases by one unit, the risk of being WML (+) (1/0.979) increases 1.021 times (p=0.014). When the year of migraine diagnosis increases by one unit, the risk of being WML (+) increases 1.178 times (p=0.029). Other parameters were not statistically significant in terms of the risk of being WML (+) (Table 3).

**Table 3:** Analyzing the risk factors affecting WMH (+) with binary logistic regression analysis WMH: white matter hyperintesity; LDL-c: low-density lipoprotein cholesterol; HDL-c: high-density lipoprotein cholesterol; NLR: neutrophil-to-lymphocyte ratio; PLR: platelet-to-lymphocyte ratio; SSI: systemic immune-inflammation index (platelet count x neutrophil count)/lymphocyte count Cox & Snell R2=0.313; Nagelkerke R2=0.423; mean ± standard deviation; frequency (%)

	Univariate		Multiple	
	OR (%95 CI)	p-value	OR (%95 CI)	p-value
Age	1.125 (1.061–1.192)	<0.001	1.075 (0.992–1.166)	0.079
LDL-c (mg/dl)	1.007 (0.996–1.018)	0.204	1.020 (0.995–1.046)	0.113
HDL-c (mg/dl)	1.033 (1.003–1.063)	0.028	1.042 (0.996–1.09)	0.076
Triglyceride (mg/dl)	0.997 (0.993–1.002)	0.287	0.979 (0.963–0.996)	0.014
Mean frequency of headache (months)	0.981 (0.933–1.032)	0.461	0.935 (0.838–1.044)	0.234
Mean disease duration (years)	1.208 (1.092–1.336)	<0.001	1.178 (1.017–1.365)	0.029
NLR	1.432 (0.928–2.21)	0.105	0.780 (0.33–1.844)	0.572
PLR	1.012 (1.002–1.022)	0.017	1.016 (0.999–1.033)	0.072
SII	1.002 (1–1.003)	0.021	-	-
Sex				
Female		Reference		
Male	0.531 (0.123–2.106)	0.368	1.382 (0.206–9.285)	0.739
Migraine type				
Episodic migraine		Reference		
Chronic migraine	0.778 (0.38–1.593)	0.492	1.674 (0.381–7.346)	0.495
Aura				
No		Reference		
Yes	0.809 (0.255–2.571)	0.720	0.674 (0.141–3.23)	0.622

A statistically significant cut-off value was found for the PLR parameter in differentiating being WML+ (p=0.044). Those with a PLR value of ≥139.05 indicate BWM +, and the sensitivity value is 45.10%, the specificity value is 78.95%, the PPV value is 58.97%, and the NPV value is 68.18% (Table 4).

**Table 4:** Determination of cut-off values for variables with ROC analysis NLR: neutrophil-to-lymphocyte ratio; PLR: platelet-to-lymphocyte ratio; SSI: systemic immune-inflammation index (platelet count × neutrophil count)/lymphocyte count

	Cut off	AUC (%95 CI)	p-value	Sensitivity (%)	Specificity (%)
NLR	---	0.558 (0.455–0.661)	0.266	---	---
PLR	≥139.05	0.606 (0.504–0.707)	0.044	45.10%	78.95%
SII	---	0.600 (0.499–0.701)	0.056	---	---

## Discussion

In this retrospective study, risk factors that may contribute to the development of WMH in migraine patients were identified according to clinical characteristics and blood tests. Our study showed that increasing age and length of migraine diagnosis were associated with the development of WMH. However, no association was observed between gender, number of migraine attacks, presence of aura, migraine type, and the development of WMH. 

Aging is independently a risk factor for the development of WMH and increases cumulatively with age [7,20]. To avoid this biased conclusion regarding the prevalence of WMH at older age, we included patients younger than 50 years in our study. As observed in previous studies, the age of migraine patients with WMH in our study was older than that of migraine patients without WMH [21]. Also, the relationship between disease duration and WMH observed in our study was compatible with studies in the literature [22-24]. This was not surprising considering both the pathogenesis of migraine and the pathogenesis considered to be effective in the development of WMH in patients with migraine. In addition, some studies in the literature have shown that the frequency of migraine attacks, severity of attacks, and the presence of a diagnosis of chronic migraine are also associated with the development of WMH [22-25]. In our study, the frequency of attacks, chronic migraine, and WMH development were also examined, and no statistically significant relationship was identified.

There are many studies indicating that migraine with aura increases the risk of cerebrovascular disease [26-29]. It causes ischemic injury secondary to hyperperfusion and hypoperfusion episodes due to cortical spreading depolarization, especially during migraine with aura [22,23]. Based on this, a relationship between aura and WMH is expected [30,31]. However, studies on this issue are controversial in the literature. Similar to our study, some studies have argued that migraine patients with and without aura have a similar risk for WMH, and another study demonstrated that the association of aura in middle-aged women predisposes to WMH, and even the cerebellum is more significantly affected in the presence of this condition [32-34].

One of the striking findings in our study was that HDL-c was found to be higher in WMH+ migraine patients. As is known, atherosclerosis starts with the oxidation of LDL-c particles in the vascular endothelium [35]. HDL-c shows protective, i.e., antioxidant and anti-inflammatory effects against oxidation caused by LDL-c [36]. In a previous study conducted by Uygur-Kucukseymen and Akca, HDL-c levels were found to be higher in migraine patients compared to the healthy control group. The potential reason for this is considered to be due to the increase in HDL-c as an antioxidant in response to the oxidative stress that occurs, especially during the attack process in migraine patients [37]. Considering that multiple cellular processes, including oxidative stress and inflammation, are involved in the pathogenesis of both migraine and aging-related WMHs, the presence of increased HDL-c, an antioxidant, may be an indicator of WMH development in migraine patients [23,38].

Studies have shown that migraine has an association with the risk of ischemic stroke [26,27,29,39]. Therefore, it may be important to anticipate the development of WMH during the follow-up of migraine patients. Parameters indicating inflammation, which is a precursor in the development of WMH and leads to oligemia, ischemia, and blood-brain barrier damage, will help in this regard. It is also important that these parameters be accessible, affordable, and practical. The studies conducted in the literature on this subject are limited. In a study conducted by Ulusoy, a correlation was observed between the monocyte/HDL-c ratio and WML [24]. In another study conducted by Mehdiyev et al., it was discussed that SII and RDW may be an indicator of the presence of WMH in migraine [40]. In another study conducted by Morkavuk et al., the relationship between NLR in migraine patients with and without WMH was examined, and a correlation was observed between WMH and NLR [41]. In the conclusions of our study, no correlation was observed between WMH, SII, and NLR, whereas PLR was found to be an independent value that can predict the development of WMH. There are various studies indicating that markers such as NLR and SII may be indicators of inflammation, whereas PLR also provides information about thrombotic/hemostatic mechanisms [42]. Therefore, the relationship between WMH and PLR observed in our study may provide insight into the pathogenesis of WMH in migraine patients, which may be related to impaired thrombotic/hemostatic mechanisms. However, it's important to remember that while numerous studies have previously demonstrated the use of hematologic parameters like NLR and SII as markers of acute and chronic inflammation in stroke, demyelinating diseases, cancer, pneumonia, and cardiac diseases, their lack of specificity for neuroinflammation-related diseases could potentially cause confusion [43-45]. These parameters should be used with caution.

Our study has several limitations. First, the number of patients in our single-center and retrospective study is limited. In particular, the scarcity of patients diagnosed with migraine with aura may have been misleading about the effects of aura on WMH. It is well known that both ergotamine and triptan may cause vasoconstriction and, therefore, may predispose to ischemic stroke [46]. Therefore, the effects of drugs administered in migraine treatment on the development of WMH should be taken into consideration. However, symptomatic or prophylactic treatment information was not available in our data and could not be evaluated.

## Conclusions

In conclusion, in our study, we examined the factors contributing to the development of WMH in migraine patients and observed the correlation between disease duration and WMH. While no correlation was observed between LDL-c and WMH, HDL-c was strikingly elevated in WML (+) patients. In addition, our study shows that the PLR ratio is a useful, easily accessible, and practical parameter to detect the development of WMH in migraine patients. Larger-scale randomized studies are needed to interpret these findings more clearly.
